# Analysis of N^6^-Methyladenosine Methylome in Adenocarcinoma of Esophagogastric Junction

**DOI:** 10.3389/fgene.2021.787800

**Published:** 2022-01-24

**Authors:** Jia-Bin Huang, Bin-Bin Hu, Rong He, Lian He, Chen Zou, Chang-Feng Man, Yu Fan

**Affiliations:** Cancer Institue, Affiliated People’s Hospital of Jiangsu University, Zhenjiang, China

**Keywords:** epigenomics, adenocarcinoma of esophagogastric junction, m^6^A methylation, diagnosis, treatment

## Abstract

**Background:** From previous studies, we found that there are more than 100 types of RNA modifications in RNA molecules. m^6^A methylation is the most common. The incidence rate of adenocarcinoma of the esophagogastric junction (AEG) at home and abroad has increased faster than that of stomach cancer at other sites in recent years. Here, we systematically analyze the modification pattern of m^6^A mRNA in adenocarcinoma at the esophagogastric junction.

**Methods:** m^6^A sequencing, RNA sequencing, and bioinformatics analysis were used to describe the m^6^A modification pattern in adenocarcinoma and normal tissues at the esophagogastric junction.

**Results:** In AEG samples, a total of 4,775 new m^6^A peaks appeared, and 3,054 peaks disappeared. The unique m6A-related genes in AEG are related to cancer-related pathways. There are hypermethylated or hypomethylated m^6^A peaks in AEG in differentially expressed mRNA transcripts.

**Conclusion:** This study preliminarily constructed the first m^6^A full transcriptome map of human AEG. This has a guiding role in revealing the mechanism of m^6^A-mediated gene expression regulation.

## Introduction

In recent years, more and more research studies are based on epigenetics. Epigenetics is a study of reversible and inheritable phenotypes. This study does not involve changes in nuclear DNA sequences (Ng and Gurdon, 2008). RNA epigenetics includes N^1^-methyladenosine (m^1^A), N^6^-methyladenosine (m^6^A), 5-methylcytidine (m^5^C), and 7-methylguanosine (m^7^G) (Zhao et al., 2017). Among them, m^6^A, discovered in the 1970s, is the most abundant internal modification of mRNA in most eukaryotes (Chen and Wong, 2020). It involves almost all stages of the life cycle, including normal and pathological processes, for example, animal development (Frye et al., 2018), gene expression regulation (Roundtree et al., 2017), and human diseases (Hsu et al., 2017). With the discovery of research, the occurrence and development of many diseases are closely related to the changes in m^6^A modification, including tumors (Li J. et al., 2021), obesity (Zhao et al., 2014), infertility (Tang et al., 2018), autoimmune diseases (Zhang Y. et al., 2019), neurological diseases (Wu et al., 2019) and so on. Desrosiers et al. (1974) found that about 0.1–0.4% of adenosine in isolated RNA is modified by m^6^A in Sox. Transcriptome-wide research reveals that m6A modifications are enriched in the 3′-untranslated regions (UTRs) near the stop codons of mRNAs and it has a consensus sequence of RRACH (R = G or A; H = A, C, or U) (Dominissini et al., 2012). m^6^A modifications are mainly mediated by “writers,” “erasers” and “reader” proteins (Liu et al., 2014). Writers traditionally include methyltransferase-like 3 and 14 proteins (METTL3 and METTL14) and their cofactors WTAP (Wilms tumor suppressor-1-associated protein) (Ping et al., 2014; Schwartz et al., 2014). METTL3 is a member of the S-adenosine-L-methionine-dependent methyltransferase family, and is the main catalytically active enzyme for m^6^A methylation modification and is highly conserved in eukaryotes (Schöller et al., 2018). METTL14 has no catalytic domain and has no enzymatic activity, but it can form a stable heterodimer complex with METTL3 (Zhang et al., 2020). Therefore, METL3 and METL14 constitute the core and structure of the complex, respectively (Wang et al., 2016). WTAP is a pre-mRNA splicing regulator with independent methylation sites. It is mainly responsible for assisting the targeting of METL3 and METL14 to nuclear sites and can specifically methylate some m^6^A sites (Zheng et al., 2019; Liu S. et al., 2020; Zhang et al., 2021). There are also new research findings, methyltransferase-like protein 16 (methyltransferase like 16, METL16) (Aoyama et al., 2020), CCCH-type zinc finger protein 13 (Zinc finger CCCH-type containing 13, ZC3H13) (Wen et al., 2018), RNA binding motif protein 15/15B (RNA binding motif protein 15/15B, RBM15/15B) (Wang T. et al., 2020) is also a component of the methyltransferase complex and can participate in m^6^A methylation modification. Demethylation is achieved by another enzyme family called demethylases (erasers), mainly including FTO and ALKBH5 (Jia et al., 2011; Zheng et al., 2013). In addition to writers and erasers, m^6^A readers also play an important role in m^6^A methylation (Shi et al., 2019). Readers which can recognize m^6^A modification contain the YT521B homology (YTH) domain (Liu et al., 2018). The YTH domain in human cells, including the YTH domain family (YTHDF1-3), YTH domain-containing 1 (YTHDC1), and YTH domain-containing 2 (YTHDC2), have conserved the m^6^A binding domain (Qin et al., 2021). Recent studies have also reported that eukaryotic initiation factor 3 (eIF3) (Liu T. et al., 2020), heterogenous nuclear ribonucleoprotein A2B1 (heterogenous nuclear ribonucleoprotein A2B1, HNRNPA2B1) (Li K. et al., 2021), insulin-like growth factor 2 messenger ribonucleic acid Binding protein 1/2/3 (insulin-like growth factor 2 mRNA binding protein 1/2/3, IGF2BP1/2/3) (Huang et al., 2018) can also be used as m^6^A reading protein. However, there are still few studies on the related mechanisms of m6A methylation.

Gastrointestinal (GI) cancers are one of the most common malignancies, accounting for more than one-fourth of the newly diagnosed cancers worldwide (more than 4 million new cases per year) (Macha et al., 2014; Zhang S. et al., 2019). Among the GI cancers, the esophagogastric junction, or esophagogastric junction (EGJ), is a special anatomical site with a remarkably high risk of adenocarcinoma (Keeney and Bauer, 2006). The incidence of adenocarcinoma of the esophagogastric junction (AEG) has been increasing both in the West and East (Steevens et al., 2010; Yamashita et al., 2017; Imamura et al., 2019). There has been much debate as to the optimal therapy for AEG, and the debate continues (Kauppila and Lagergren, 2016). Here, we demonstrate the presence of m^6^A modification in adenocarcinoma of esophagogastric junction *via* m^6^A-methylated RNA immunoprecipitation (MeRIP) sequencing (MeRIP-seq), a powerful strategy for transcriptome-wide mapping of RNA modifications in mRNAs (Lin et al., 2018). We report transcriptome m^6^A profiling in adenocarcinoma of esophagogastric junction samples and the tumor-adjacent normal tissues for the first time.

## Materials and Methods

### Tissue Collection

Four pairs of matched adenocarcinoma of the esophagogastric junction and para-cancerous tissue samples were derived from adenocarcinoma of esophagogastric junction patients who underwent radical surgery in Affiliated People’s Hospital of Jiangsu University, Zhenjiang from July 2018 to November 2018. Adenocarcinoma of esophagogastric junction tissue was excised from the central part of the tumor; the paired paracancerous tissue was taken from normal tissue that was more than 5 cm from the edge of the tumor and had a negative margin. The collection process for all tissue samples was completed within 30 min after the tumor was isolated. The fresh tissue was cut into tissue pieces of about 5 mm in diameter and quickly placed in a sterilized cryotube and stored in an ultra-low temperature freezer at −80°C.

### RNA Preparation

For each group, four biological replicates were selected, of which every two were combined into one. Then, the total RNA of tissue was extracted using TRIzol reagent (Invitrogen Corporation, CA, United States) in accordance with the manufacturer’s instructions. The Ribo-Zero rRNA Removal Kit (Illumina, Inc., CA, United States) was used to reduce the ribosomal RNA content of total RNAs. Then, the RNA was chemically fragmented into fragments of about 100 nucleotides in length using fragmentation buffer (Illumina, Inc.).

### High-Throughput m^6^A and mRNA Sequencing

**T**otal RNA was harvested from tissue samples and underwent a quality control (QC) process using NanoDrop ND-1000 (Thermo Fisher Scientific, MA, United States). High-throughput m^6^A and mRNA sequencing were performed by Cloudseq Biotech, Inc. (Shanghai, China) according to the published procedure with slight modifications. Briefly, fragmented RNA was incubated with anti-m^6^A polyclonal antibody (Synaptic Systems, 202003, Goettingen, Germany) in IP, immunoprecipitation buffer at 4°C for 2 h (Wang H.-F. et al., 2020). The mixture was then immunoprecipitated by incubation with protein-A beads (Thermo Fisher Scientific) at 4°C for an additional 2 h. Then, bound RNA was eluted from the beads with N^6^-methyladenosine (Berry & Associates, PR3732, Dexter, United States) in IP buffer and then extracted with Trizol reagent (Thermo Fisher Scientific) according to the manufacturer’s instruction. Purified RNA was used for RNA-seq library generation with NEBNextR Ultra™ RNA Library Prep Kit (New England Biolabs, MA, United States). Both the input samples without immunoprecipitation and the m^6^A IP samples were subjected to 150 bp paired-end sequencing on Illumina HiSeq sequencer, Illumina, CA, United States.

### Sequencing Data Analysis

Paired-end reads were harvested from Illumina HiSeq 4000 sequencer and were quality controlled by Q30. After 3′adapter-trimming and low-quality reads were removed using cutadapt software (v1.9.3) (Kechin et al., 2017), the high-quality clean reads of all libraries were aligned to the reference genome (UCSC HG19) by Hisat2 software (v2.0.4) (Kim et al., 2015). For m^6^A sequencing, methylated sites on RNAs (m^6^A peaks) were identified by MACS software (Zhang et al., 2008). Differentially methylated sites were identified by diffReps (Shen et al., 2013). For mRNA sequencing, raw counts were identifiedby HTSeq software (v0.9.1) and normalized by edgeR software. Differentially expressed mRNAs were identified by *p*-value and fold change. Gene ontology (Geistlinger et al., 2021) and pathway enrichment analysis (Tian et al., 2005) were performed based on the differentially methylated protein coding genes and differentially expressed mRNAs.

### Gene-Specific MeRIP-qPCR Validation

Three genes with differentially methylated sites according to m^6^A-seq were tested by reverse transcription RT-qPCR. A portion of fragmented RNA was saved as the input control. The rested RNA was incubated with anti-m^6^A antibody-coupled beads. The m^6^A-containing RNAs were then immunoprecipitated and eluted from the beads.

The following are the gene-specific qPCR primers:

PLCG2:Forward:AGCTTAACCTTCAACCTGTGTG, Reverse:AAGATAGCTTTTACGGTTGGGT

TPR:Forward:TGCTTTTGGAGAAACTAGAGAACA, Reverse:TGGCGTTTCAAAATTGGTGC

DVL1:Forward:AGCTGCTTCTGTGTAAATGCT, Reverse:GTCCCATAAAATTAAACGCTTTT

GAPDH:Forward:GGCCTCCAAGGAGTAAGACC, Reverse:AGGGGAGATTCAGTGTGGTG

### Statistical Analysis

Data from three or more independent experiments were presented as the mean ± standard deviation (SD). Statistical analysis was done using SPSS 22.0 and GraphPad Prism 5.0 software. Paired Student t-tests were performed between cancer and adjacent normal samples. One-way analysis of variance was used to access the differences among three or more groups. Differences with *p* < 0.05 were defined as the threshold for significance.

## Results

### Characterization of m^6^A Methylation in Adenocarcinoma of Esophagogastric Junction

**FIGURE 1:**
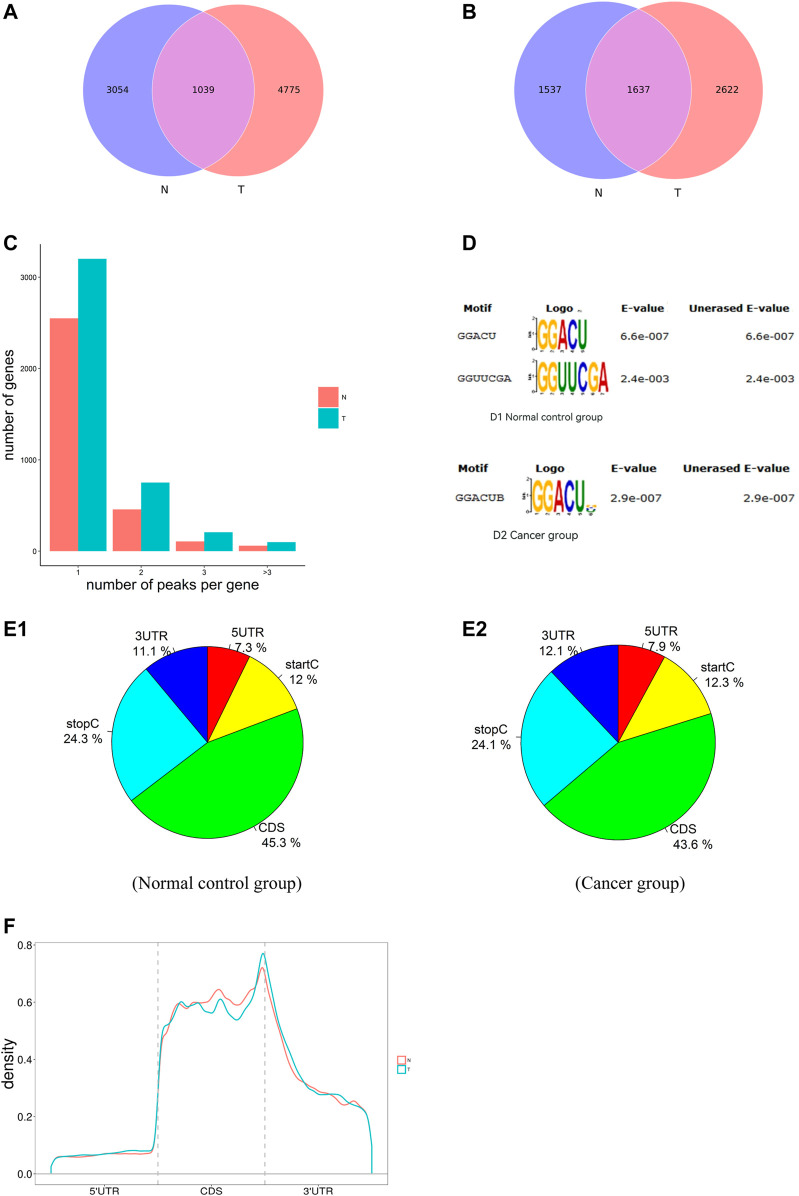
Characteristics of m^6^A methylation in AEG. **(A)** The conserved/specific m^6^A methylation peak. **(B)** The conserved/specific m^6^A methylation genes. **(C)** the distribution of m^6^A-modified peaks per gene. **(D)** The RRACH conserved sequence motif for m^6^A-containing peak regions. **(E)** The percentage of m^6^A methylated peaks in transcripts. Each transcript is divided into five parts: 5′UTRs, CDS, 3′UTRs, start codon, stop codon. **(F)** Accumulation of m^6^A peaks along with transcripts. m^6^A, N^6^-methyladenosine.

Human AEG tissues versus tumor-adjacent normal tissues from four patients were selected for transcriptome-wide m^6^A-sequencing (m^6^A-seq) and RNA-sequencing (RNA-seq) assays. The original sequencing data IP is 50011084-103480210; Input is 49555862-60311666. After preprocessing of the original data (to remove the connector, to remove low-quality reads, to obtain high-quality clean reads), IP is 11574526-76461856; Input is 49100868-60160794 (Table 1). m^6^A is known to be a relatively abundant mRNA modification (Chen and Wong, 2020). In general, a total of 5,814 m^6^A peaks were identified by model-based analysis of ChIP-seq (MACS) (Nilsen, 2014) in the Ca group, representing transcripts of 4,259 genes. In the tumor-adjacent NC group, 4,093 m^6^A peaks were identified, which correspond with transcripts of 3,174 genes. Among them, 1,039 m^6^A peaks (only ∼12% of all peaks in cancer and normal groups) were detected within both adenocarcinomas of esophagogastric junction tissues and normal tissues. The low percentage of overlapping m^6^A peaks within mRNAs suggests differences in the m^6^A patterns between two groups (Figures 1A,B). By analyzing the distribution of m^6^A-modified peaks per gene, we found the majority of genes had one to three m^6^A modified sites, while a relatively small number of genes contain more (Figure 1C).

**TABLE 1:** Sequencing reads.

Sample name	Raw reads	Clean reads
737511T.IP	51,034,044	29,314,686
737789T.IP	50,011,084	11,574,526
738156T.IP	91,721,102	42,114,984
739939T.IP	73,896,158	38,570,024
737511N.IP	67,019,630	31,154,202
737789N.IP	103,480,210	76,461,856
738156N.IP	60,176,662	36,498,028
739939N.IP	101,048,022	54,723,832
737511T.Input	55,202,914	54,242,640
737789T.Input	60,311,666	60,160,794
738156T.Input	54,079,092	53,918,834
739939T.Input	50,681,654	50,594,076
737511N.Input	55,340,698	55,193,614
737789N.Input	55,598,832	55,423,884
738156N.Input	52,120,622	51,107,154
739939N.Input	49,555,862	49,100,868

#### Motif Analysis of RNA Methylation Region

RNA methylation and demethylation begin with the motif binding of various binding proteins to methylation sites. A motif is essentially a sequence pattern of nucleic acids with biological significance, and these RNA methylation-related enzymes recognize and bind to these motifs, thereby affecting gene expression (Wang et al., 2019). To determine if the m^6^A peaks that we identified contained the m^6^A consensus sequence of RRACH (where R represents purine, A is m^6^A, and H is a non-guanine base). The m^6^A methylomes were further mapped by De reme software. The sequence of the top 300 peaks with the largest enrichment factor of each group (50 bp on each side of the vertex) was taken, and the sequence of these peaks was scanned using De reme to find a meaningful motif sequence. Deme (Bailey, 2011) motif analysis of methylated mRNAs revealed the existence of some motifs containing the consensus sequences (RRACH) of m^6^A modification (Figure 1D).

Then, we analyzed the distribution of m^6^A in the whole transcriptome of Ca and NC samples. We determined the distribution of m^6^A reads along with transcripts in the m^6^A-IP and non-IP (input) samples, respectively. Both total and unique m^6^A peaks from the two groups were analyzed. m^6^A peaks were divided into transcription start codon (start c), 5′UTR, coding sequence (CDS), stop codon (stop c) and 3′UTR according to their locations in RNA transcripts. Intriguingly, In general, the m^6^A peaks were enriched in the vicinity of CDS, the stop codon, and the start codon (Figure 1E). As shown in Figure 1E, about 70% of m^6^A peaks are located in the intergenic region; more than 60% of them are located near the CDS region and stop codon region; while about 30% of m^6^A peaks are located in the 5′UTR, start codon, and 3′UTR region. The distribution trend of the two tissues is highly similar, which indicates that the classical recognition sequence of m^6^A methylation is conserved in human tissues.

To better understand the distribution of m^6^A peaks in the whole mRNA, we divided each m^6^A modified mRNA into three regions: 5′UTR, CDS, and 3′UTR, and calculated the distribution proportion of these three regions. It can be seen from Figure 1F that the curve of the whole region of 5′UTR changes very gently until the distribution proportion of m^6^A peaks increases rapidly near the start codon. On the whole, peaks in the CDS area are highly enriched, however, the curve change in the middle of the CDS area is also relatively gentle, which shows that the distribution of the peaks in the middle of CDS is relatively uniform. But there is a peak of m^6^A peaks near the stop codon, which indicates that the distribution of m^6^A peaks increases rapidly when the end of CDS is near the stop codon. In the 3′UTR region, m^6^A peaks decrease rapidly from the beginning of the 3′UTR to the 3 ′ ends (Figure 1F).

### Differential Methylated Genes Analysis

**FIGURE 2:**
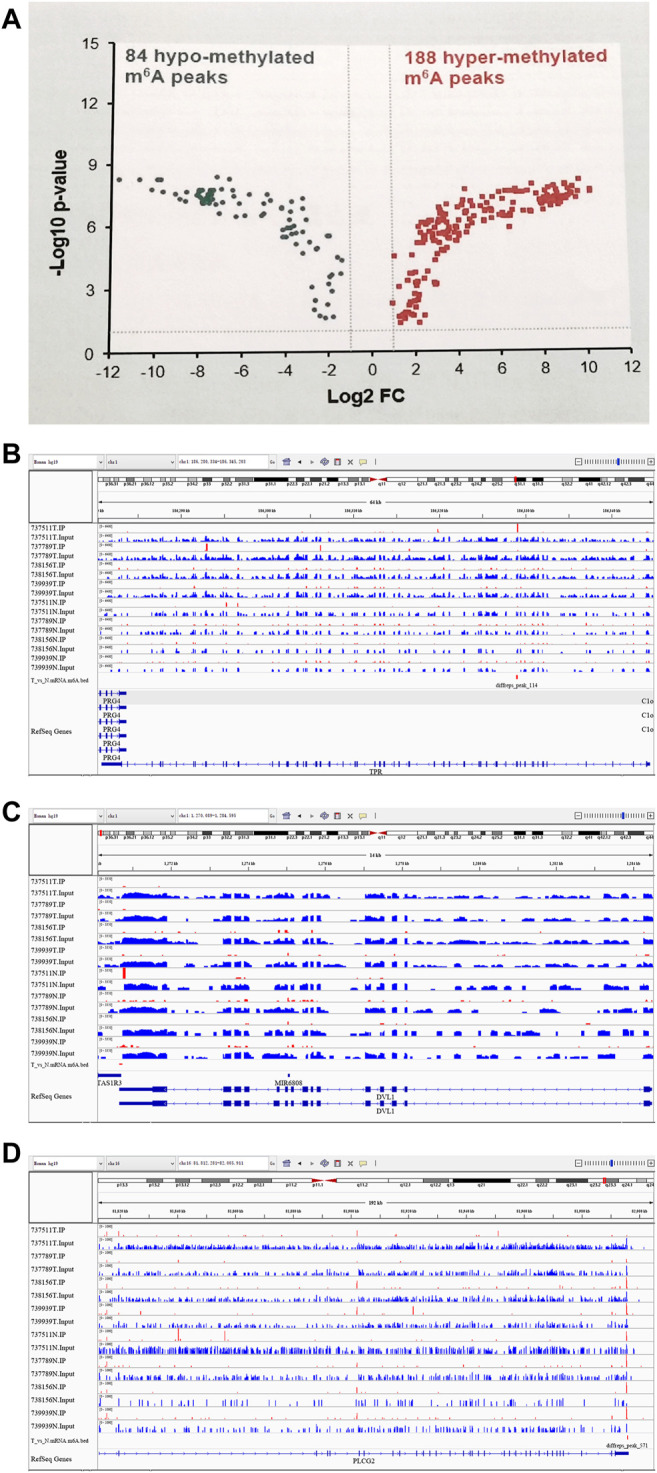
Differentially methylated N^6^-methyladenosine peaks in the tumer and control groups. **(A)** 188 m^6^A hypermethylation sites and 84 m^6^A hypomethylation sites. **(B)** A representative up-methylated gene in the tumor group relative to the control group. **(C,D)** two representative down-methylated genes in the tumor group relative to the control group.

The abundance of the m^6^A peaks between NC and Ca samples was compared. Among the 1,039 m^6^A peaks detected in bothsamples, a total of 272 differentially methylated sites were chosen for further study. Among them, there are 188 m^6^A hypermethylation sites and 84 m^6^A hypomethylation sites (Figure 2A). According to the Integrative Genomics Viewer (IGV) software, the hypermethylation gene TPR and hypomethylation genes PLCG2 and DVL1 were displayed (Figures 2B–D).

### Identification of Differentially Expressed Genes in AEG by RNA-Seq

**FIGURE 3:**
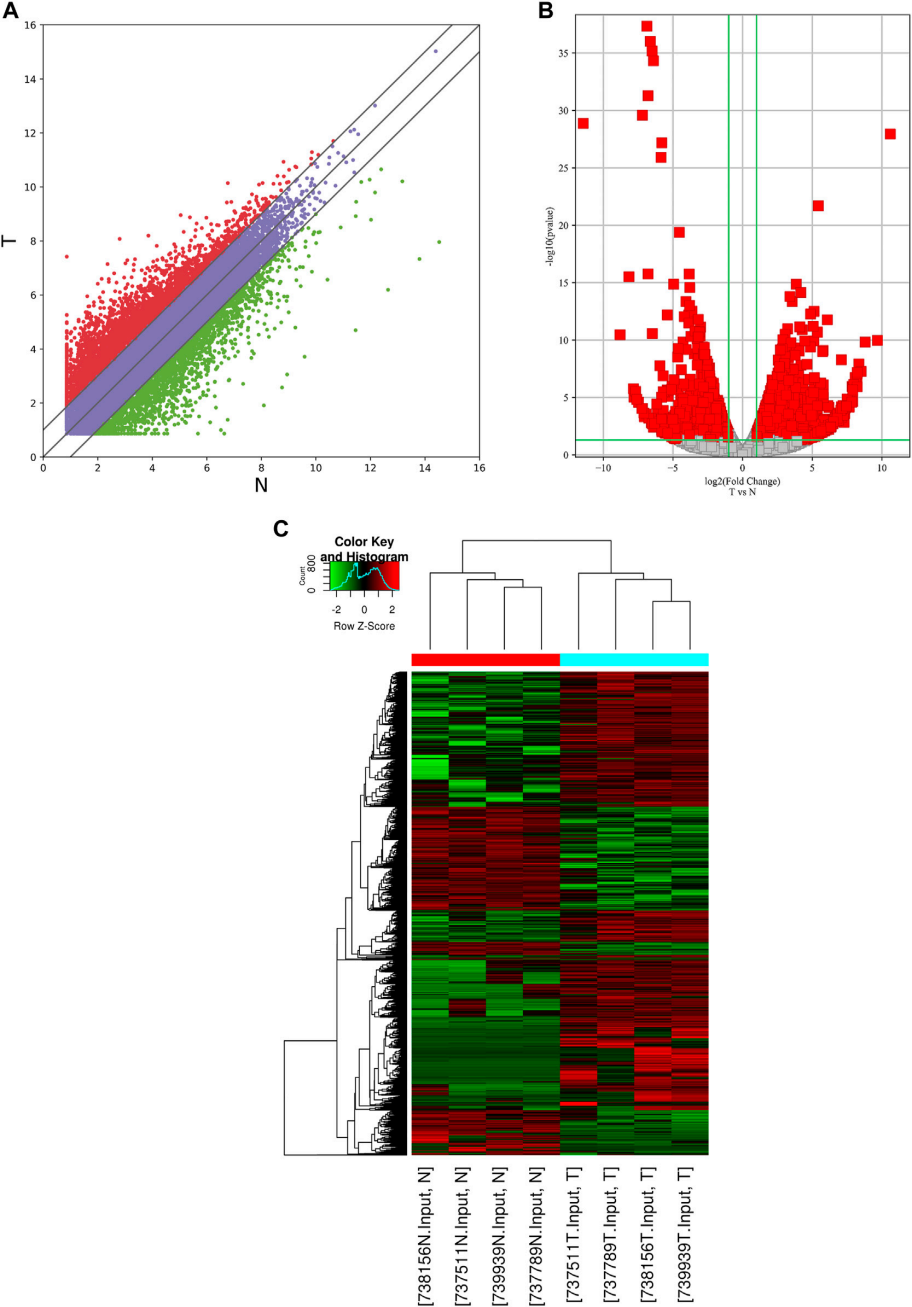
Identification of differentially expressed genes in AEG by RNA-seq. **(A)** the scatter plot of the RNA-seq data. **(B)** Volcano Plot of the RNA-seq data. **(C)** Heat map of RNA-seq data of Ca samples and adjacent normal tissues. Red and Green indicate the upregulation and downregulation of mRNAs, respectively.

In the RNA-seq dataset (m^6^A-seq input library), we discovered that the global mRNA expression patterns between AEG samples and adjacent normal tissues were significantly different. We calculated gene expression to assign differentially expressed genes (DE genes) of the two tissues. Of the 20,308 mRNAs we have identified, 3,069 were significantly different, while 17,239 were not. Among them, 2,032 is up-expressed and 1,037 is down-expressed (fold change > 2, *p* < 0.05). The volcano Plot, scatter plot, and the hierarchical clustering of the RNA-seq data were shown in Figures 3A–C.

### Conjoint Analysis of m^6^A-RNA Binding Protein Immunoprecipitation (RIP)-Seq and RNA-Seq Data of AEG and Normal Samples

**FIGURE 4:**
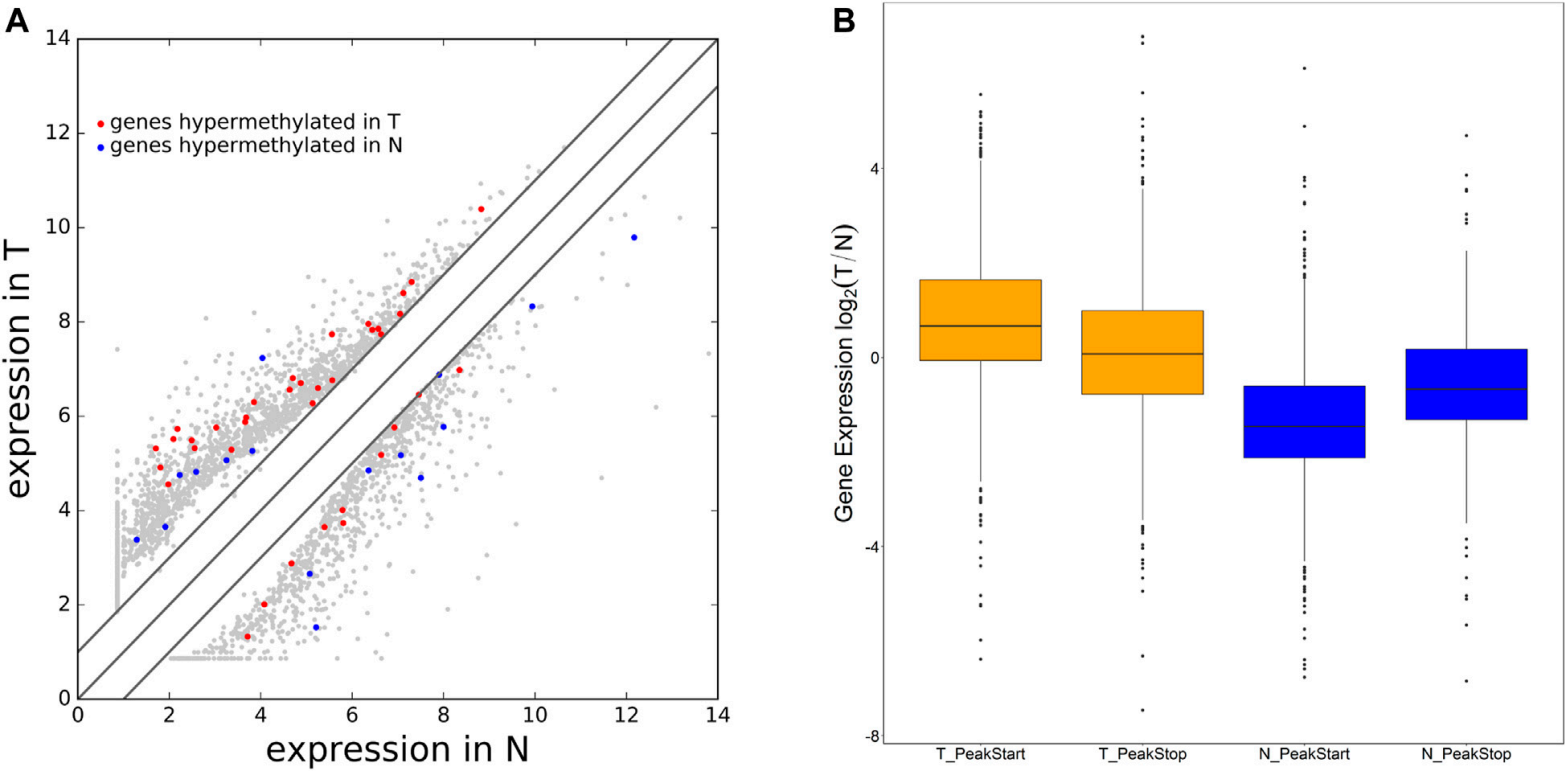
Differential expression mRNA harboring differentially methylated N^6^-methyladenosine sites. **(A)** Differentially expressed mRNA in AEG and matched normal tissue. **(B)** The ratio of mRNA expression levels in two samples containing tumor/normal-specific m^6^A peaks.

By cross-analysis of the m^6^A-Seq and RNA-seq data, we studied the relationship between the expression level of the m^6^A modified gene and its mRNA. Using RNA-seq, we found that there were 3,069 differential expression mRNAs, of which 2032 is up-expressed and 1,037 is down-expressed (fold change >2, *p* < 0.05). In 158 hyper-methylated mRNAs detected by m^6^A-Seq, we found thirty targets with upregulated mRNA transcripts (fold change > 2, *p* < 0.05), namely “hyper-up” (Table 2). The expression of 9 hypermethylation mRNA was downregulated (fold change > 2, *p* < 0.05), namely “hyper-down” (Table 3). In the contrast, 7 of 77 genes with hypomethylated m^6^A sites showed upregulated mRNA transcripts (fold change > 2, *p* < 0.05), namely, “hypo-up” (Table 4), and 11 of 77 genes with hypomethylated m^6^A sites showed downregulated mRNA transcripts (fold change > 2, *p* < 0.05), namely, “hypo-down” (Table 5). Notably, the numbers of “hyper-up” and “hypo-down” genes were more than those of “hyper-down” and “hypo-up” genes. To describe the relationship between differential m^6^A modification and its mRNA expression, we plotted a scatter plot. The results show that m^6^A modifications tend to have a positive correlation of mRNA expression in AEG. However, further analysis of the underlying mechanisms is needed (Figure 4A).

**TABLE 2:** Hyper-up gene.

Gene name	Pattern	Chromosome	m^6^A level change					mRNA level change		
			Peak region	Peak start	Peak end	Fold change	*p*-value	Strand	Fold change	*p*-value
MYL12B	Hyper-up	chr18	3′UTR	3278181	3278282	589.5333	4.04E-08	+	2.396340637	0.014228
TRIM69	Hyper-up	chr15	Exonic	45059581	45059960	503.3184	4.62E-08	+	4.361604634	0.01379
TPR	Hyper-up	chr1	Exonic	186000000	186000000	449.3787	4.63E-08	−	2.168355042	0.011414
OLFM4	Hyper-up	chr13	Exonic	53624161	53624380	438.9512	6.89E-08	+	41.40861373	2.09E-11
SNX1	Hyper-up	chr15	3′UTR	64433121	64433400	384.0109	6.36E-08	+	2.273696986	0.020023
FTSJ3	Hyper-up	chr17	3′UTR	61896792	61897000	195.3784	9.49E-09	−	2.83970881	0.000421
THEM6	Hyper-up	chr8	Exonic	144000000	144000000	113.0642	1.61E-08	+	8.357769668	0.000168
DGCR6	Hyper-up	chr22	Exonic	18899052	18899340	105.5676	8.06E-08	+	14.36709026	0.000414
PDAP1	Hyper-up	chr7	Exonic	98997925	98998047	52.41343	0.000000425	−	2.939484153	0.000234
DNAJC21	Hyper-up	chr5	Exonic	34954657	34954960	49.44994	0.000000153	+	2.263359888	0.043718
DIAPH3	Hyper-up	chr13	Exonic	60435387	60435640	42.13218	0.000000179	−	11.87765394	0.005924
ESF1	Hyper-up	chr20	Exonic	13698014	13698161	29.20853	0.000000346	−	10.77001236	0.000268
PHLPP2	Hyper-up	chr16	3′UTR	71681201	71681560	26.70845	0.00000006	−	4.971430367	0.001788
ASPM	Hyper-up	chr1	Exonic	197000000	197000000	21.8376	0.00000021	−	19.67930001	0.00000146
ERCC1	Hyper-up	chr19	Exonic	45981993	45982086	16.93339	0.000000127	−	3.083275918	0.000306
C2orf15	Hyper-up	chr2	Exonic	99766945	99767400	15.76066	0.00000106	+	3.179251174	0.007309
NONO	Hyper-up	chrX	Exonic	70516700	70516897	15.57156	0.00000206	+	2.190048774	0.008746
SMC6	Hyper-up	chr2	Exonic	17899343	17899490	12.53989	0.00000364	−	18.74389666	0.0000171
TPX2	Hyper-up	chr20	Exonic	30380621	30380633	9.823938	0.000000183	+	3.704793164	0.000704
ITGB1	Hyper-up	chr10	Exonic	33218749	33218960	9.433681	0.00000235	−	2.644302086	0.001906
CLCC1	Hyper-up	chr1	Exonic	109000000	109000000	8.576991	0.0000246	−	2.662759259	0.003414
CENPE	Hyper-up	chr4	Exonic	104000000	104000000	7.295365	0.000000566	−	10.56417353	0.007515
CENPF	Hyper-up	chr1	Exonic	215000000	215000000	6.658635	0.000000557	+	4.678360385	0.00000188
ELF1	Hyper-up	chr13	Exonic	41507441	41507760	6.604386	0.000000654	−	2.597993822	0.010944
FEN1	Hyper-up	chr11	3′UTR	61564361	61564714	5.984716	0.000000896	+	9.734534556	0.001119
VRK2	Hyper-up	chr2	Exonic	58373450	58373609	5.662622	0.00000371	+	4.759549413	0.00018
POLE3	Hyper-up	chr9	Exonic	116000000	116000000	5.036067	0.00000204	−	4.962024986	0.000619
SMC3	Hyper-up	chr10	Exonic	112000000	112000000	3.85501	0.000192	+	4.625598352	0.004154
NCL	Hyper-up	chr2	Exonic	232000000	232000000	3.772817	0.0000264	−	2.980307104	0.000137
NCL	Hyper-up	chr2	Exonic	232000000	232000000	3.330097	0.000445	−	2.980307104	0.000137

**TABLE 3:** Hyper-down gene.

Gene name	Pattern	Chromosome	m^6^A level change					mRNA level change		
			Peak region	Peak start	Peak end	Fold change	*p*-value	Strand	Fold change	*p*-value
SERTAD4	Hyper-down	chr1	Exonic	210000000	210000000	913.1	5.73E-09	+	26.69078811	0.006686
UTRN	Hyper-down	chr6	Exonic	145000000	145000000	454.6408	2.88E-08	+	2.596053293	0.001236
MED12L	Hyper-down	chr3	Exonic	151000000	151000000	74.36694	5.11E-08	+	7.028418024	0.036049
FAT4	Hyper-down	chr4	Exonic	126000000	126000000	67.88037	0.000000305	+	3.732908122	0.024065
SPECC1	Hyper-down	chr17	Exonic	20135070	20135144	27.71836	0.000000773	+	2.791750475	0.007659
RTL1	Hyper-down	chr14	Exonic	101000000	101000000	11.87251	8.89E-08	−	4.938866594	0.000164
TBC1D9B	Hyper-down	chr5	Exonic	179000000	179000000	4.061465	0.001852	−	2.012892324	0.027099
F13A1	Hyper-down	chr6	Exonic	6251043	6251162	2.535	0.048738	−	4.559260999	0.04251
OSBPL1A	Hyper-down	chr18	Exonic	21750290	21750417	2.18732	0.0000285	−	3.859703434	0.009948

**TABLE 4:** Hypo-up gene.

Gene name	Pattern	Chromosome	m^6^A level change					mRNA level change		
			Peak region	Peak start	Peak end	Fold change	*p*-value	Strand	Fold change	*p*-value
CENPE	Hypo-up	chr4	Exonic	104000000	104000000	185.5455	2.98E-08	−	7.145663	0.007515
ZNF697	Hypo-up	chr1	Exonic	120000000	120000000	170.5844	0.000000018	−	4.600914	0.031955
RPS27A	Hypo-up	chr2	3′UTR	55462741	55462960	5.496705	0.0000121	+	3.890766	0.048552
MTRNR2L3	Hypo-up	chr20	3′UTR	55933521	55933840	92.85938	4.97E-09	−	6.133057	0.013268
TOP2A	Hypo-up	chr17	Exonic	38551700	38551791	376.6434	6.58E-08	−	34.88758	3.49E-09
ZNF678	Hypo-up	chr1	Exonic	228000000	228000000	8.666667	4.72E-08	+	5.339058	0.020853
ARL5B	Hypo-up	chr10	3′UTR	18964181	18964500	156.4742	4.77E-08	+	5.166949	0.023021

**TABLE 5:** Hypo-down gene.

Gene name	Pattern	Chromosome	m^6^A level change					mRNA level change		
			Peak region	Peak start	Peak end	Fold change	*p*-value	Strand	Fold change	*p*-value
FAM46C	Hypo-down	chr1	Exonic	118000000	118000000	4.347762	0.027232	+	26.56062	0.000000255
C7	Hypo-down	chr5	Exonic	40936439	40936587	217.58	6.58E-08	+	20.4862	0.00000601
ITIH5	Hypo-down	chr10	3′UTR	7601231	7601400	215.1854	5.97E-08	-	10.17475	0.001424
PLCG2	Hypo-down	chr16	3′UTR	81995801	81996000	120.633	1.65E-08	+	6.860429	0.008813
SETBP1	Hypo-down	chr18	Exonic	42533001	42533305	13.46909	4.88E-07	+	13.83856	0.000199
TSC22D3	Hypo-down	chrX	Exonic	107000000	107000000	3.483142	0.001349	−	24.92234	0.000000597
MYH11	Hypo-down	chr16	Exonic	15832421	15832540	15.24643	3.46E-06	−	30.97305	2.62E-08
MYH11	Hypo-down	chr16	Exonic	15831305	15831477	16.42946	3.49E-06	−	30.97305	2.62E-08
JUN	Hypo-down	chr1	Exonic	59247581	59247780	10.93321	2.17E-06	−	15.46234	0.0000842
DVL1	Hypo-down	chr1	Exonic	1270655	1270740	358.0816	2.39E-08	−	5.618537	0.017771
PGC	Hypo-down	chr6	3′UTR	41704448	41704460	141.6648	9.11E-08	−	160.2992	9.73E-37

We were wondering whether the location of m^6^A peaks in mRNA transcripts was associated with gene expression levels. To further explore how m^6^A modification affects mRNA expression, we divided the gene containing m^6^A sites into PeakStart category (m^6^A peaks around start codon) and PeakStop (m^6^A peaks around stop codon). The results showed that the PeakStart category had higher overall expression levels (Figure 4B, note that the expression ratio of the tumor/normal gene is shown).

### Results of Bioinformatics Analysis

**FIGURE 5:**
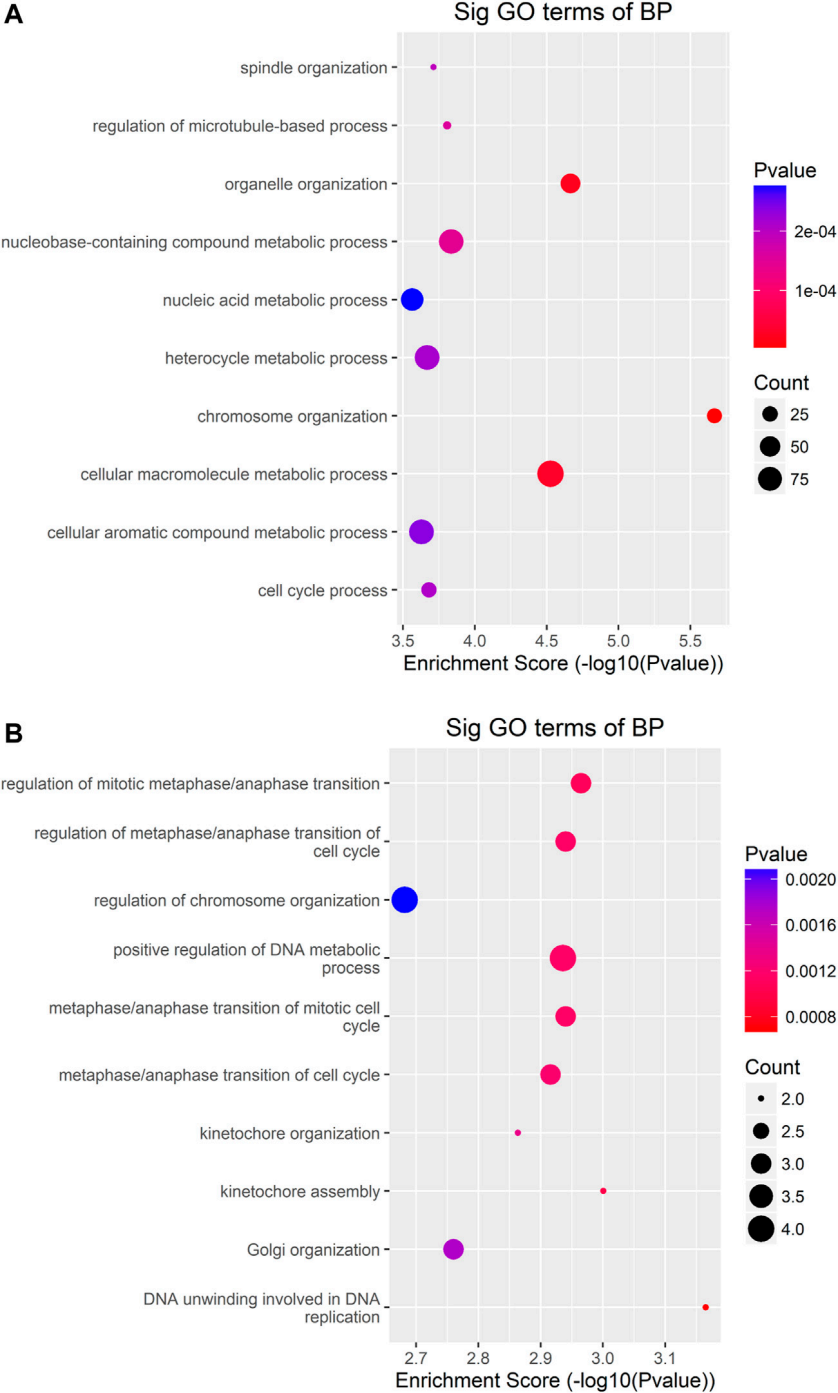
GO-enrichment analysis for differentially methylated mRNAs **(A)** The top ten gene ontology terms of biological processes were significantly enriched for the up-methylated genes. **(B)** The top ten gene ontology terms of biological process significantly enriched for down-methylated genes.

**FIGURE 6:**
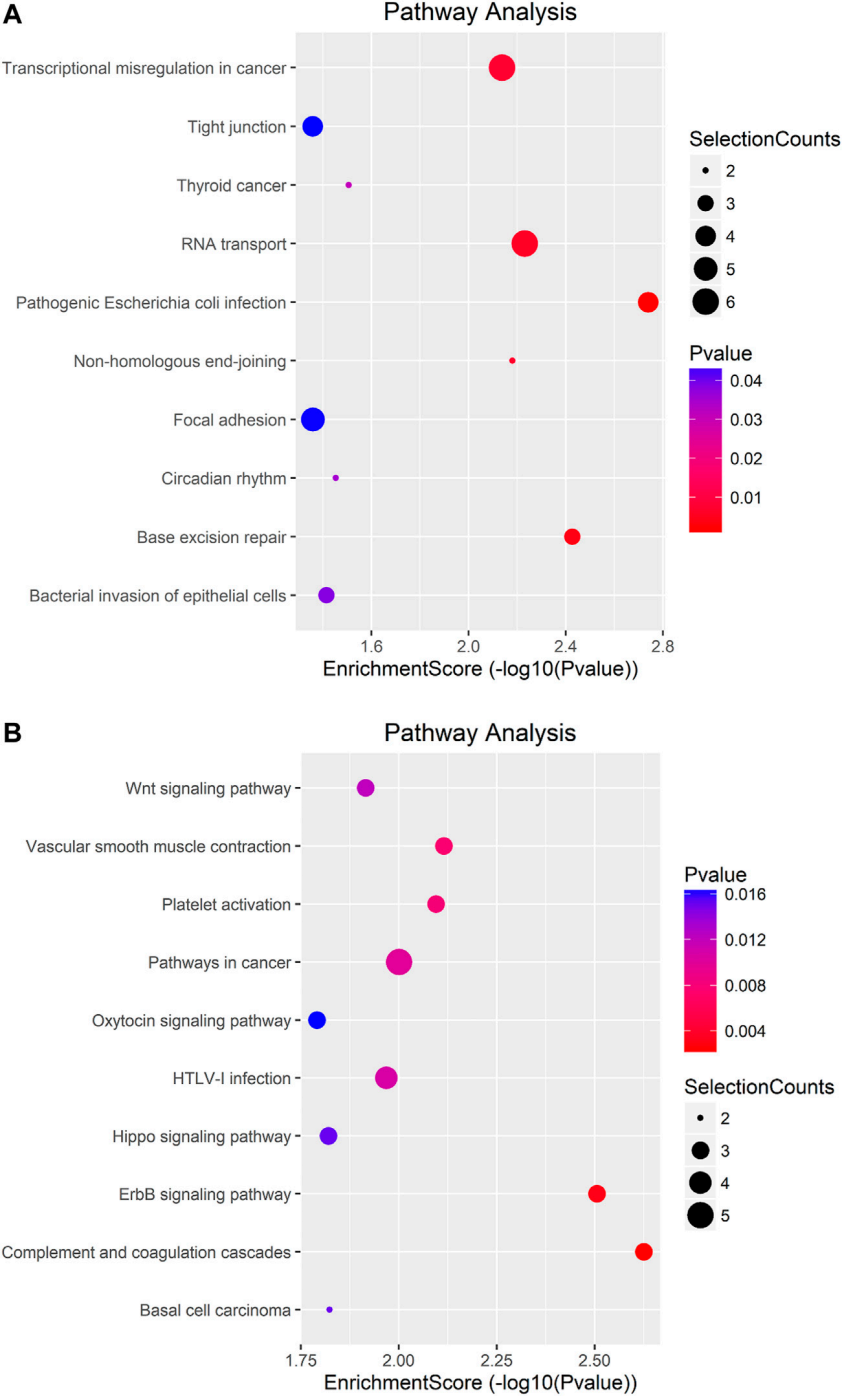
Pathway analysis of mRNAs harboring differentially methylated N^6^-methyladenosine sites. **(A)** Bubble Plot showing the top ten enrichment scores of the significant enrichment pathway for the hyper-methylated genes. **(B)** Bubble Plot showing the top ten enrichment scores of the significant enrichment pathways of the hypo-methylated genes.

To uncover the functions of m^6^A modification in AEG tissues, mRNAs containing DMMSs were selected for GO (gene ontology) enrichment analysis and KEGG pathway analysis. Differently, m^6^A methylation genes are mainly involved in cell DNA metabolism and cell cycle process (Figures 5A,B). Pathway analysis revealed that mRNAs with hypermethylated and hypomethylated m^6^A sites were enriched in many pathways involved in cancer pathogenesis, including Pathways in cancer, Basal cell carcinoma, Wnt signaling pathway, HTLV-I infection, ErbB signaling pathway (Figures 6A,B).

**FIGURE 7:**
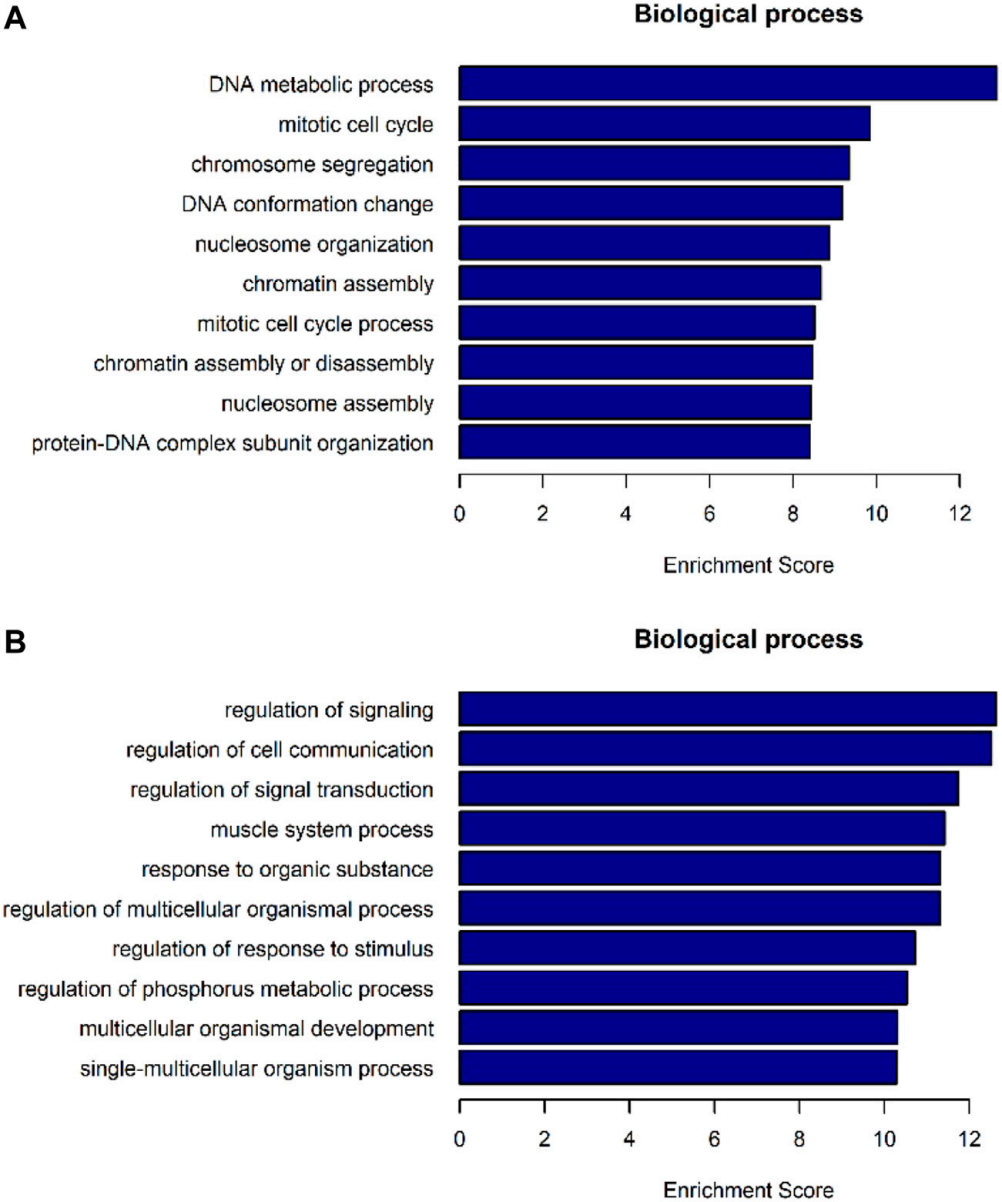
GO-enrichment analysis for differentially expressed mRNAs. **(A)** Top 10 biological processes of mRNA enrichment up-regulated by differences. **(B)** Top 10 biological processes of mRNA enrichment were differentially down-regulated.

**FIGURE 8:**
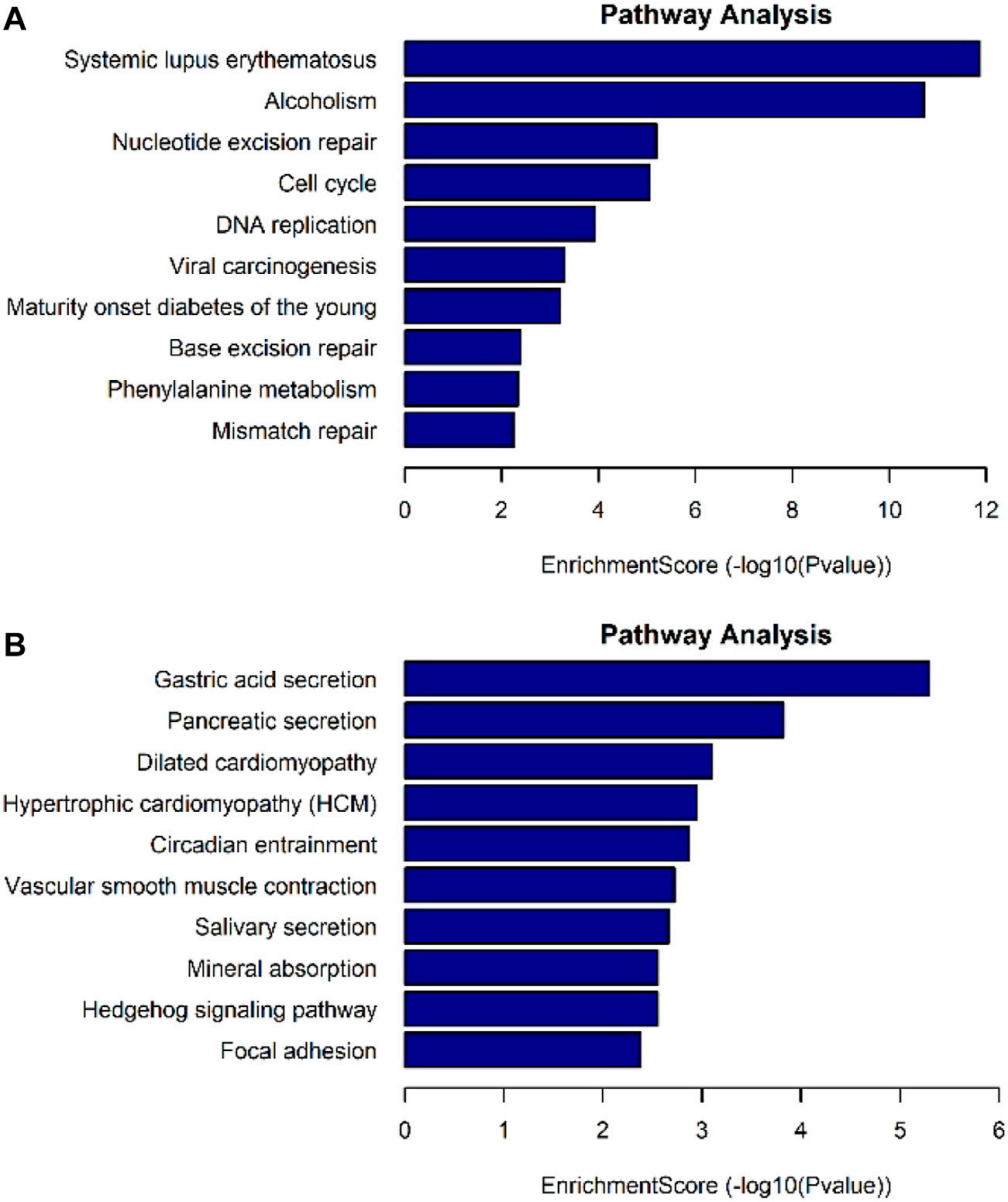
Pathway analysis for differentially expressed mRNAs. **(A)** Up-regulated mRNAs related pathway analysis. **(B)** Down-regulated mRNAs related pathway analysis.

The differentially expressed genes were selected for ingenuity gene ontology and pathway analysis. It revealed that differentially expressed genes were significantly enriched in biological processes involving DNA metabolic process, cell cycle process (Figures 7A,B). Moreover, pathway analysis showed that nucleotide excision repair, cell cycle, and DNA replication were significantly altered in AEG samples (Figures 8A,B).

### The Results of the Preliminary Screening Were Further Verified by mRNA qPCR and MeRIP-qPCR

**FIGURE 9:**
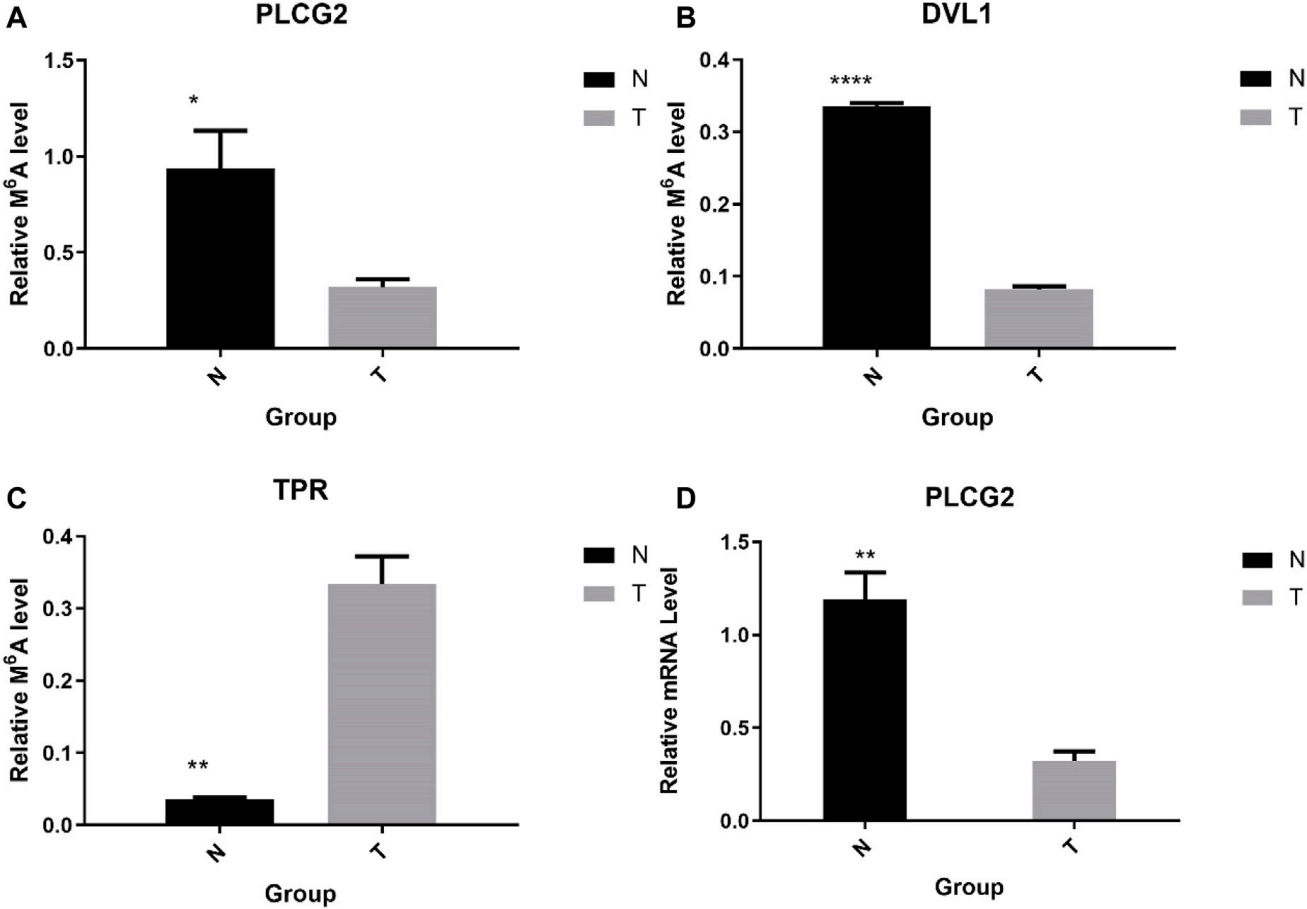
Gene-specific m^6^A qPCR assays and detection of global m^6^A levels. **(A–C)** Gene-specific m^6^A qPCR validation of m^6^A level changes of three representative hyper-methylated or hypo-methylated genes in the normal control group and the AEG group samples. **(D)** Relative mRNA level of representative genes was measured by real-time PCR in normal control group and AEG group samples.

To further confirm the results of our m^6^A-seq data, we conducted gene-specific MeRIP-qPCR assays for several hypermethylated and hypomethylated genes (TPR, DVL1, and PLCG2). TPR is hypermethylated, but PLCG2, DVL1 are hypomethylated (Table 6 shows the initial detection of methylation of three genes). MeRIP-qPCR results showed that TPR was hypermethylated, while DVL1, PLCG2 were hypomethylated (Figures 9A–C, *p* < 0.05). We observed the same m^6^A-level changes in three out of the three genes (100%), demonstrating the reliability of our transcriptome-wide m^6^A-seq data.

**TABLE 6:** Initial detection of methylation of three genes.

mRNA	Fold change	Methylation level	Chromosome localization
PLCG2	120.633	Down	chr16
TPR	449.3787	Up	chr1
DVL1	358.0816	Down	chr1

Sequentially, we verified the expression of mRNA by qPCR. We chose PLCG2 as the validation gene. The above results show that the expression of PLCG2 is downregulated, and the fold change is 2.935315743 (Table 7). And after verification by qPCR, the results indicate that PLCG2 is downregulated (Figure 9D, *p* < 0.05). The results of qPCR and MeRIP-qPCR elaborated that the PLCG2 acting as a hypomethylated gene, its expression was down-expressed.

**TABLE 7:** Expression of PLCG2 obtained by initial screening.

mRNA	Fold change	Expression level	Chromosome localization
PLCG2	2.9534157	Down	chr16

The results of qPCR and MeRIP-qPCR showed that the Melcurve Plots of GAPDH and three mRNAs were single peaks, and the inflection points of each Amplification Plot were obvious, the overall parallelism was good, and the baseline was smooth without rising, indicating that the PCR amplification product specificity, amplification efficiency.

### The Results of the Conservation of m^6^A Validated on the ConsRM Online Platform

**FIGURE 10:**
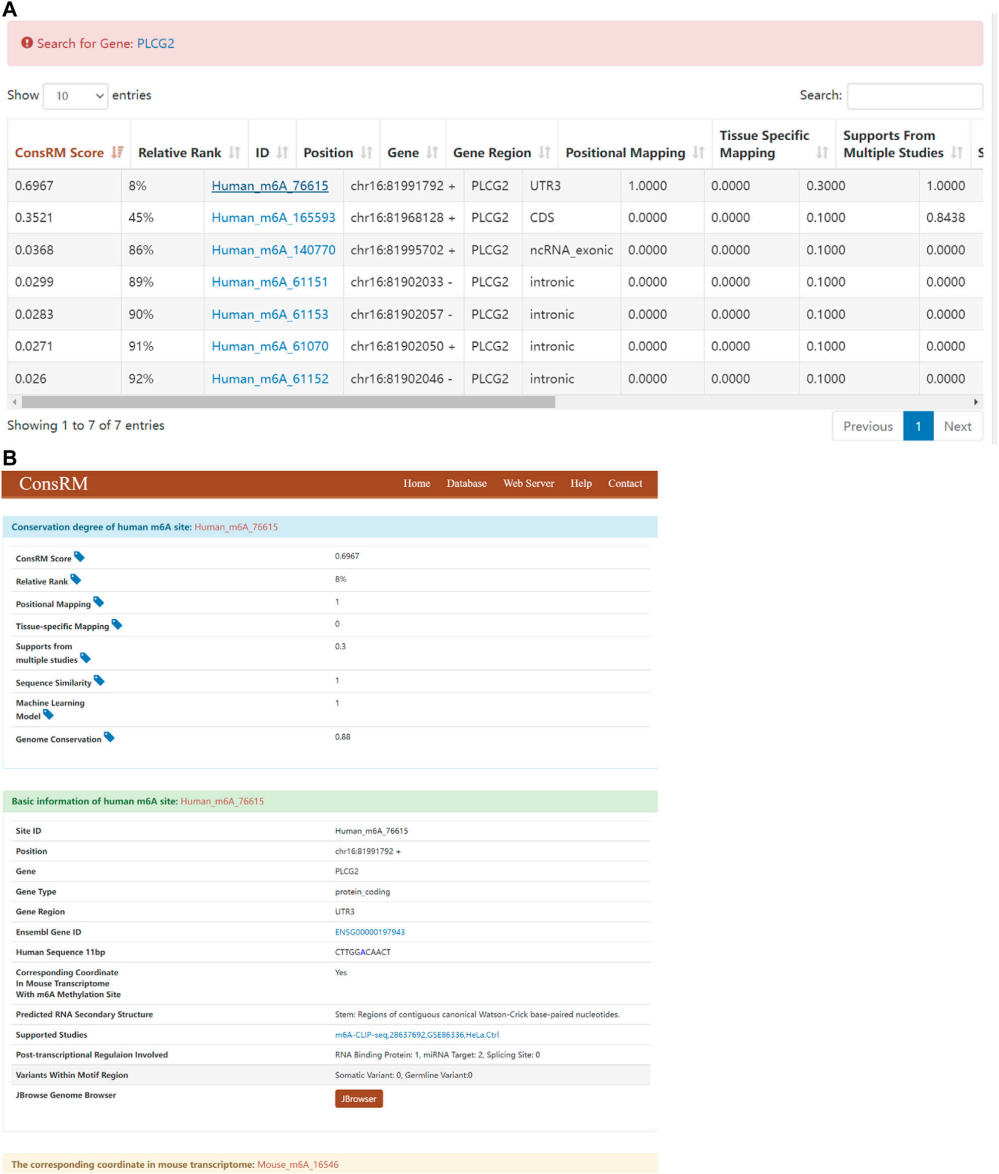
Results of ConsRM online platform. **(A)** Search for “PLCG2” returns 7 m^6^A sites. **(B)** The detailed conservation metrics and other regulatory information of the most conserved m^6^A site on PLCG2.

The recent studies have been proven the m^6^A modification in evolution conservation (Miao et al., 2021; Song et al., 2021), thus, the conservation could be considered in our analysis. Taking PLCG2 as an example, Search for “PLCG2” returns 7 m^6^A sites located on the PLCG2 transcripts on the ConsRM online platform. One of them are highly conserved, among the top 8% most conserved m^6^A sites (Figure 10A). In addition, we can also see that the Gene Region where the most conserved m^6^A site of PLCG2 is located is 3′UTR, which is consistent with our experimental results. Its post-transcriptional regulation involves one RNA binding protein site and two miRNA Targets (Figure 10B).

## Discussion

Recent technological advances in high-throughput sequencing in combination with antibody enrichment of modifications have accelerated the detection of distribution and abundance for m^6^A methylation at the transcriptome-wide level (Meyer et al., 2012; Strong et al., 2015). The discovery of m^6^A demethylases indicates that m^6^A methylation of mRNA is a reversible and dynamic process with regulatory functions (Jia et al., 2013; Nilsen, 2014).

In recent years, more and more studies have been conducted on the components of m^6^A writers, erasers, and readers in cancer. Ma et al. (2017) revealed an important role of METTL14 in tumor metastasis and provided a fresh view of m^6^A modification in tumor progression. Zhang J. et al. (2019) indicated a novel mechanism by which ALKBH5 promotes GC invasion and metastasis by demethylating the lncRNA NEAT1. Tang et al. (2019) found that FTO is essential for the proliferation of pancreatic cancer cells. In our study, we found that the expression level of YTHDF3 was up-regulated (FC > 2, *p* < 0.05, Table 8), but there was no previous study on the expression level of YTHDF3 in adenocarcinoma of the esophagogastric junction. But limited by the size of our research sample, more research is needed to further explore. But in HCC, Zhou et al. (2019) found that the expression level of YTHDF3 was upregulated.

**TABLE 8:** Expression of m^6^A methylated regulator.

Gene	Chromosome localization	Fold change	Expression level	*p* value
ZC3H13	chr13	1.732127499	Up	0.051304693
RBM15	chr1	1.263324488	Up	0.557236768
KIAA1429	chr8	1.112162637	Down	0.806428469
METTL3	chr14	1.320213898	Up	0.390769705
METTL14	chr4	1.368793596	Up	0.712905503
WTAP	chr6	1.687160095	Up	0.099719678
FTO	chr16	1.017027554	Up	0.975420319
ALKBH5	chr17	1.493330599	Down	0.349730675
YTHDF1	chr20	1.428774733	Up	0.348060186
YTHDF2	chr1	1.497757976	Up	0.501900856
YTHDF3	chr8	2.79836485	Up	0.049556735
YTHDC1	chr4	1.130674806	Up	0.70423955
YTHDC2	chr5	1.887976428	Down	0.353781994
HNRNPC	chr14	1.911794749	Up	0.138018091

In this study, we illustrated global m^6^A modification patterns in AEG samples vs. tumor-adjacent normal tissues, analyzing gene expression and cancer-related pathways modulated by abnormal m^6^A RNA modifications. From previous studies, we know that m^6^A modified nucleotides are widely distributed in animal tissues, including the heart, liver, kidney, brain, and lung (Dominissini et al., 2012; Meyer et al., 2012). We figured out that the m^6^A modification pattern in AEG samples was distinct from that of normal controls, with a higher total m^6^A level and 1,721 more m^6^A peaks identified in the Ca group. By analyzing the differently methylated transcripts, cancer-related biological processes and pathways were significantly enriched, indicating the relationship between abnormal m^6^A modifications and AEG pathogenesis. Such global change of m^6^A modification profiles could result from the abnormal expression of key m^6^A enzymes. Nevertheless, only ∼12% of all peaks were detected within both adenocarcinomas of esophagogastric junction tissues and normal tissues. So there are differences between the two kinds of tissues. By analyzing the distribution of m^6^A-modified peaks per gene, we found the majority of genes had one to three m^6^A modified sites, while a relatively small number of genes contain more. Similarly, Chen et al. (2020) found that the majority of genes (6268/8526) had one to three m^6^A modified sites. Using MeRIP-Seq technology, we discovered that the m^6^A peak is abundant near the CDS and the stop codon, followed by the start codon and the 3′ UTR. However dominant m^6^A enrichment near stop codons and 3′UTRs is shown in most of mammal mRNA mammal as previously reported (Dominissini et al., 2012), and this m^6^A distributing type may represent the typical m^6^A topological pattern in most of the mature mRNA (Meyer et al., 2012; Batista et al., 2014). The extensively higher m6A signals at the stop codon or 3′UTRs may be responsible for RNA stability, signaling for transport, and translocation (Niu et al., 2013; Wang X. et al., 2014). As recently reported, m^6^A sites in plants are enriched around stop codons within 3′UTRs, start codons, and 5′UTRs (Li et al., 2014; Luo et al., 2014; Wang X. et al., 2014). In order to better understand the distribution of m^6^A peaks in the whole mRNA, we divided each m^6^A modified mRNA into three regions: 5′UTR, CDs and 3′UTR, and calculated the distribution proportion of these three regions. We can conclude that the distribution of m^6^A peaks in the CDS region is increased and there is a summit in the m^6^A peak near the stop codon of CDS. But in plants, Csepany et al. (1990) found that there was another minor summit of m^6^A peaks at positions near the start codon of CDS both in callus and leaf.

The consensus motif sequence RRACH has previously been shown to be over represented in m^6^A motif regions (Wei and Moss, 1977; Harper et al., 1990) and also further been identified in some high throughput m^6^A RNA sequencing databases (Luo et al., 2014; Wan et al., 2015). Therefore, in our current study, we successfully identified common motifs in the AEG transcriptome. Interestingly, Csepany et al. (1990) failed to find the common RRACH sequence in the m^6^A motif region of rice, but another different motif sequence was enriched by MEME and HOMER.

We additionally analyzed the relationship between methylation genes and their expression levels. Combined analysis of our m^6^A-seq and mRNA-seq data revealed 55 genes in the Ca group, which have differently methylated m^6^A sites along with significant changes of mRNA abundance compared with the NC group (fold change > 2, *p* < 0.05). It indicated that m^6^A modifications tend to have a positive correlation of mRNAs expression in AEG. Similar to our results, Luo et al. (2019) found that compared to hypomethylated genes, the expression of hypermethylated mRNAs tended to be upregulated in the HFD group. But some studies have come to different conclusions (Niu et al., 2013; Schwartz et al., 2014). In addition, we found that the overall expression level of methylated genes near the start codon was higher. Luo et al. (2019) also stated that genes in the PeakStart category possess higher overall expression levels. However, in the study of Luo, the enrichment of m^6^A around the start codon is obvious. Different from the results of Luo, there is no obvious enrichment of m^6^A around the start codon in our study. Recently, it has been found that the main function of m^6^A is to mediate the degradation of mRNA in mammalian cells (Batista et al., 2014; Wang Y. et al., 2014; Liu et al., 2014).

In combination with various databases and preliminary screening results, we selected three typical mRNAs for further verification. For example, PLCG2 could promote proliferation through inactivating ERK and NF-κB pathway (Ma et al., 2019), p38 MAPK and JNK MAPK pathways (Chen et al., 2018a), PKCδ-mediated JNK1/2 signaling pathway (Chen et al., 2018b). The TPR contributes to the organization of the nuclear lamina and in cooperation with lamins guards the interphase assembly of nuclear pore complexes (Fišerová et al., 2019). Consistent with the results of the initial screening, TPR was hyper-methylated; DVL1, PLCG2 were hypomethylated. As a hypomethylated gene, the expression of PLCG2 was downregulated. It is further possible to conclude that hypomethylated genes are more prone to low expression. This is similar to the results that m^6^A modifications tend to have a positive correlation of mRNAs expression in AEG. The relationship between gene methylation and gene expression requires further investigation.

In our study, the human AEG transcriptome modification profile was proposed for the first time and differentially expressed mRNA transcripts were identified through hypermethylation and hypomethylation m^6^A modification. This may help to further study the mechanism of m^6^A-mediated gene expression regulation. It is possible to develop new AEG therapeutic strategies by regulating m^6^A methylation transcripts or m^6^A-related genes. However, this study still has some limitations: 1) the m^6^A-Atlas (Tang et al., 2021) database is currently the human genome database containing the most known modification sites. Our research only selected three typical mRNAs for further verification. In the future, we will further select more novel methylated genes in AEG to compare with methylation data in m^6^A-Atlas, and gradually improve related research. 2) The RMVar (Luo et al., 2021) and RMDisease (Chen et al., 2021) show the potential association between the mutations with m^6^A. In future work, we will further explore the role of m^6^A-related mutations in adenocarcinoma of the esophagogastric junction, look for related mechanisms, and find therapeutic targets Point, provide a theoretical basis for the precision treatment of AEG.

## Conclusion

This study preliminarily constructed the first m^6^A full transcriptome map of human AEG. This has a guiding role in revealing the mechanism of m^6^A-mediated gene expression regulation.

## Data Availability

The datasets presented in this study can be found in online repositories. The names of the repository/repositories and accession number(s) can be found below: https://www.ncbi.nlm.nih.gov/geo/GSE189874.

## Abbreviations

AEG, adenocarcinoma of esophagogastric junction; ALKBH5, a-ketoglutarate-dependent dioxygenase alkB homolog 5; eIF3, eukaryotic initiation factor 3; FTO, obesity-associated protein; HNRNPA2B1, heterogenous nuclear ribonucleoprotein A2B1; IGF2BP1/2/3, insulin-like growth factor 2 mRNA binding protein 1/2/3; m^6^A, N^6^-methyladenosine; METTL3, methyltransferase-like3; METTL14, methyltransferase-like 14; METTL16, methyltransferase like 16; RBM15/15B, RNA binding motif protein 15/15B; WTAP, Wilms tumor suppressor-1-associated protein; YTHDC1, YTH domain-containing 1; YTHDC2, YTH domain-containing 2; YTHDF1, YTH N6-methyladenosine RNA binding protein 1; YTHDF2, YTH N6-methyladenosine RNA binding protein 2; YTHDF3, YTH N6-methyladenosine RNA binding protein 3; ZC3H13, Zinc finger CCCH-type containing 13.
